# Clinical features and management of lymphoepithelial cyst

**DOI:** 10.1515/med-2023-0872

**Published:** 2023-12-07

**Authors:** Ning Cui, Wenhao Su, Jixiang Zhang, Zhongyin Zhou

**Affiliations:** Department of Gastroenterology, Renmin Hospital of Wuhan University, Wuhan, China

**Keywords:** benign lesion, asymptomatic, EUS-FNA, surgical treatment, medical cost

## Abstract

The aim of the study was to analyze clinical features of lymphoepithelial cyst (LEC) to make a more comprehensive and deeper understanding of it. We retrospectively analyzed the hospital records of 201 patients who were diagnosed by pathology results. Clinical characteristics like demographic profiles, lesion characteristics, therapeutic schedule, and associated costs were analyzed. Patient’s age ranged from 17 to 83 years old (52.6 ± 14.3, 120 males and 81 females). There were 12 cases of pancreatic LEC, 48 of oral LEC, and 141 of parotid LEC. Single lesion was found to be more than multiple lesions (147:54, 73.1%:26.9%). The majority of patients was primarily diagnosed by imaging test and endoscopy (171, 85.1%). All patients were finally confirmed by pathology results. Different treatment plans were selected according to personal situation, including dynamic observation (21, 10.5%), non-surgical treatment (24, 11.9%), and surgical treatment (156, 77.6%). No recurrence was found in surgical treatment patients for up to 24 months follow-up. To sum up, LEC is a rare and benign lesion, which is mostly located at parotid and oral, rarely located at pancreas. No typical symptoms could be found. EUS-FNA could be a reliable way to obtain pathological diagnosis. LEC could be cured by surgical resection with no recurrence.

## 1 Introduction

Lymphoepithelial cyst (LEC) is a clinical rare and benign lesion. It was first reported by Luchtrath et al. in 1985 and officially named by Truong et al. in 1987, which was originally described as “a cystic lesion filled with keratinoids, lined with mature horns.” In recent decades, only few reports were made and the clinical data of some patients were incomplete, it is important to carry out a more comprehensive research for LECs. LEC could be found in parotid, salivary gland, oral, thyroid, and pancreas [1,2]. Pancreatic LEC (PLEC) accounts for only 5% of all cystic diseases of the pancreas. Accompanied by the widespread usage of computerized tomography (CT) and magnetic resonance imaging (MRI) in clinic, the diagnostic rate of LEC is increasing year by year [3]. However, due to the lack of specific clinical symptom and specificity of imageological examination, it is difficult to make an accurate early diagnosis of LEC as well as select a personalized and optimal treatment [4]. The reason that we study its clinical features is because we need to understand the disease more comprehensively and deeply, to avoid clinical misdiagnosis and missed diagnosis so that optimal strategies could be made for the patients. Our study retrospectively analyzed the hospitalized patients who were diagnosed LEC by pathology results, which was designed to deepen the understanding of LEC and provide a better basis for clinical work and future research.

## 2 Methods

Our study retrospectively analyzed the hospitalized patients from January 2015 to December 2022, who were diagnosed LEC by pathology results obtained from the surgical resection specimens. Totally, 201 patients met the criteria. Demographic data were collected as age, gender, and occupation. Clinical characteristics like clinical manifestation, biochemical results, lesion location, size, imaging characteristics, therapeutic schedule, pathology results, associated costs, and follow-up were analyzed as well. All details of ethical approval and human rights of this study were approved by the Institutional Patient Care and Data Use Committee of Wuhan University.

## 3 Results

Demographic data: Patient’s age ranged from 17 to 83 years old (52.6 ± 14.3). Gender ratio indicated that men take a higher proportion than women (120, 59.7%: 81, 40.3%). More than half of the patients (129, 64.2%) were unoccupied. Majority lived in urban area of the country (147, 73.1%) (Table 1).

**Table 1:** Demographic data of LECs

Demographic data	
Age (year)	52.6 ± 14.3 (17–83)
**Gender**	
Male	120 (59.7%)
Female	81 (40.3%)
**Occupation**	
Occupied	72 (35.8%)
Unoccupied	129 (64.2%)
**Residence**	
Urban	147 (73.1%)
Rural	54 (26.9%)

**Figure 1:**
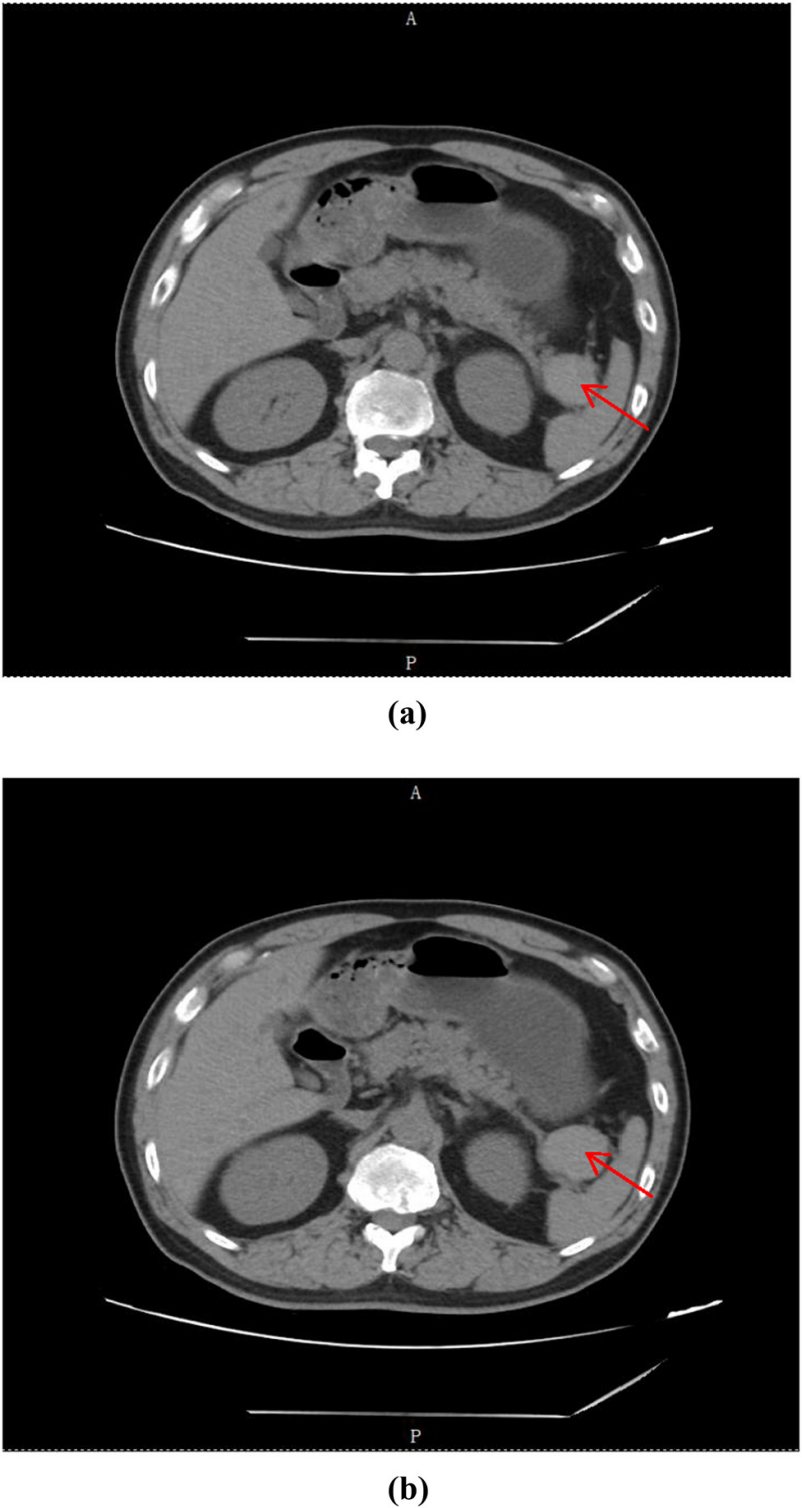
(a, b) CT scan of the abdominal space occupying lesion. The red arrow shows nearly-circular soft tissue density lesion as 88HU located at the spleen and stomach clearance.

**Figure 2:**
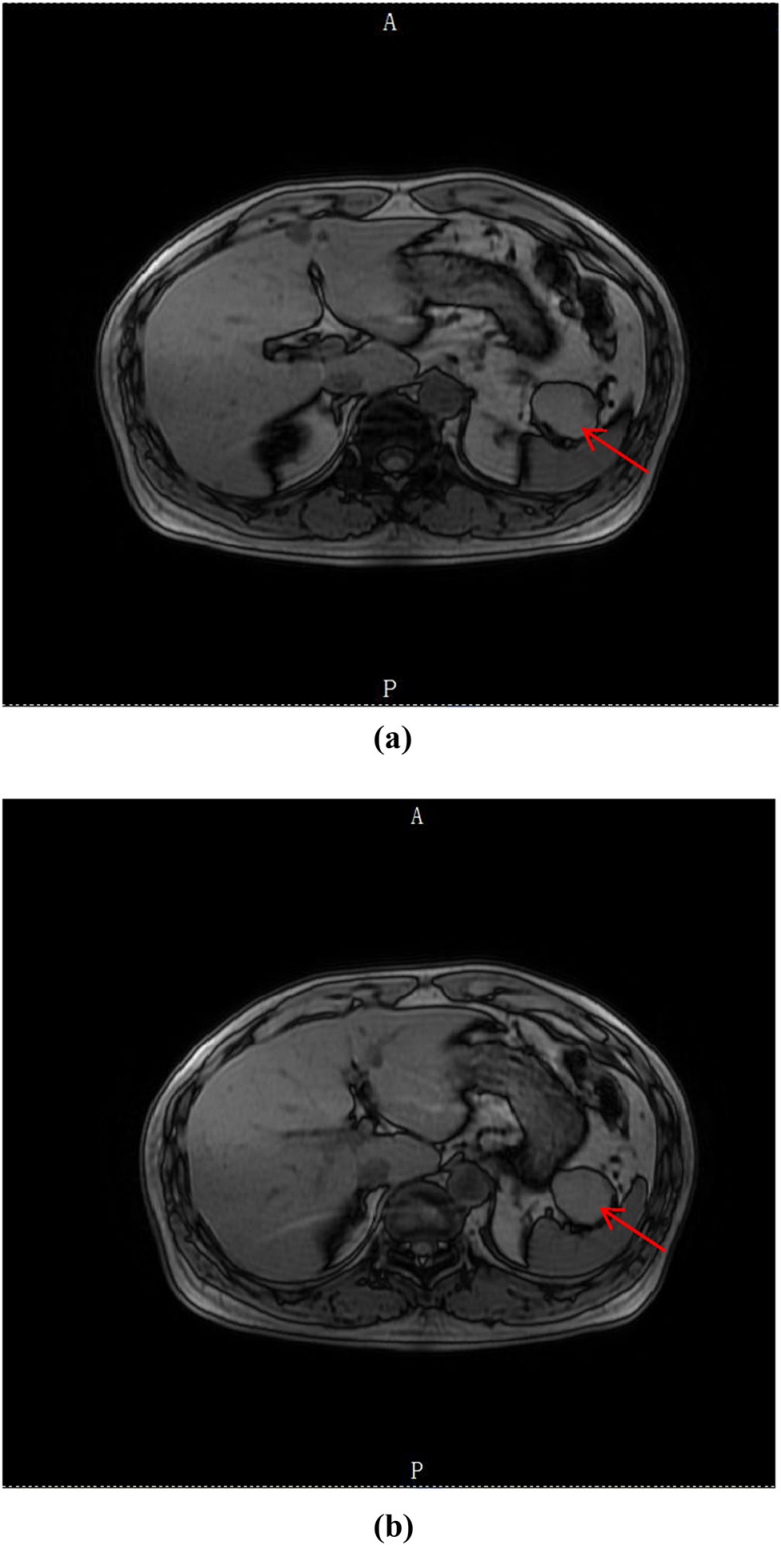
(a, b) MRI scan of the abdominal space occupying lesion. The red arrow shows nearly-circular mass with equal T1 and short T2 signal.

Clinical characteristics: Among all patients, most of them were asymptomatic (174, 86.6%). Moreover, majority of biochemical tests indicated normal level (171, 85.1%), minority showed increased level of CEA/CA125/CA199 (30, 14.9%). There were 141 cases of parotid LEC, 48 cases of oral LEC, and 12 cases of PLEC (141, 70.1%; 48, 23.9%; 12, 6.0%). The lesion size was divided into three groups: <2 cm (117, 58.2%), 2–5 cm (75, 37.3%), and >5 cm (9, 4.5%). In addition, single lesion was found to be more than multiple lesions (186, 92.5%: 15, 7.5%). All patients were primarily diagnosed by imaging tests including CT/MRI/PET-CT (201, 100%), as shown in Figures 1 and 2.

**Figure 3:**
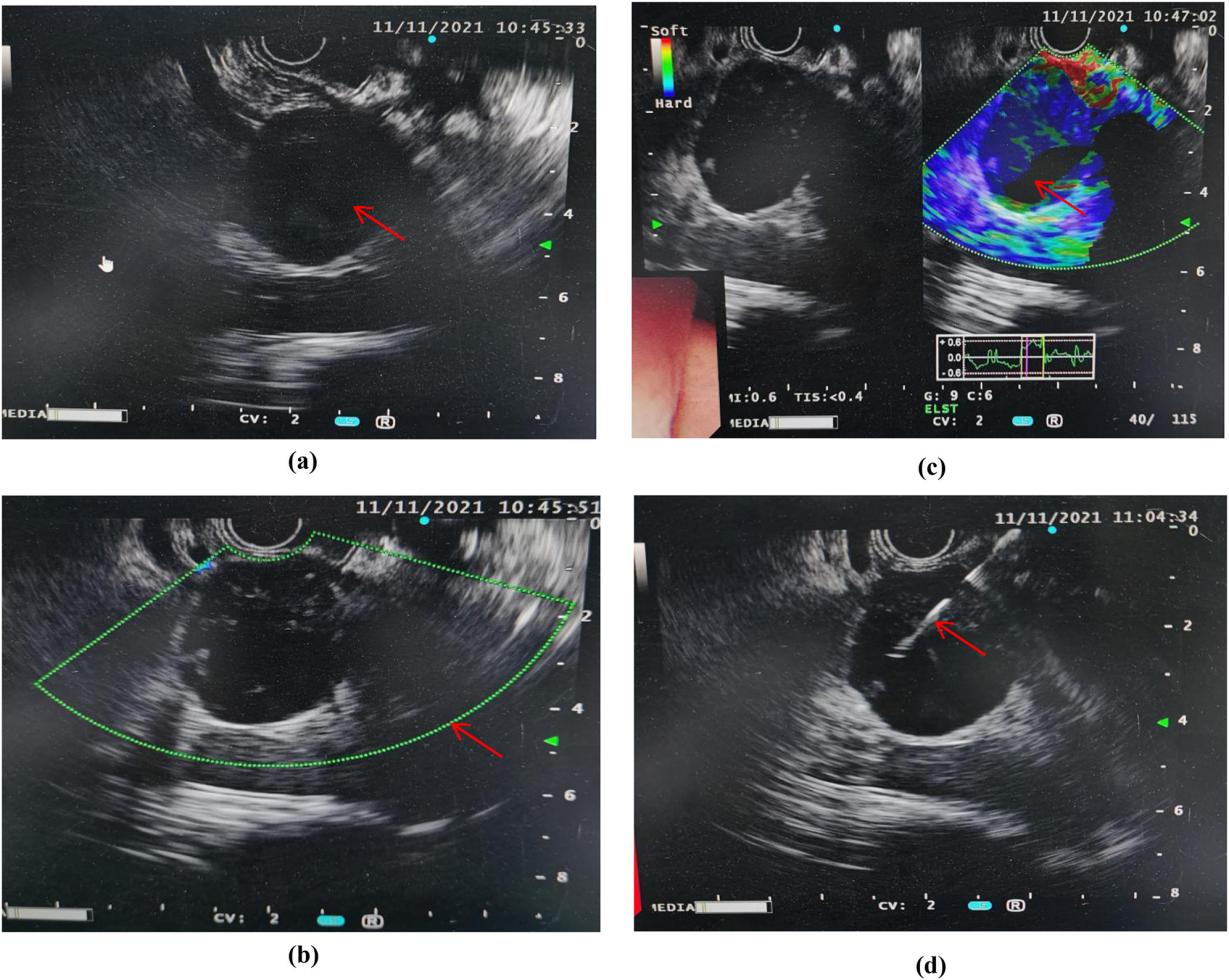
EUS and EUS-FNA operation of LEC: (a) red arrow indicates the lesion manifestation of LEC under EUS, (b) red arrow indicates that there was few blood flow signal inside the lesion, (c) red arrow indicates green, blue, and some colorless area of elastography under EUS, and (d) red arrow indicates the form of fine needle during EUS-FNA operation.

Some patients took a further step of endoscopy ultrasound (EUS) or EUS guided fine needle aspiration (EUS/EUS-FNA, 12, 6.0%), as shown in Figure 3.

**Figure 4:**
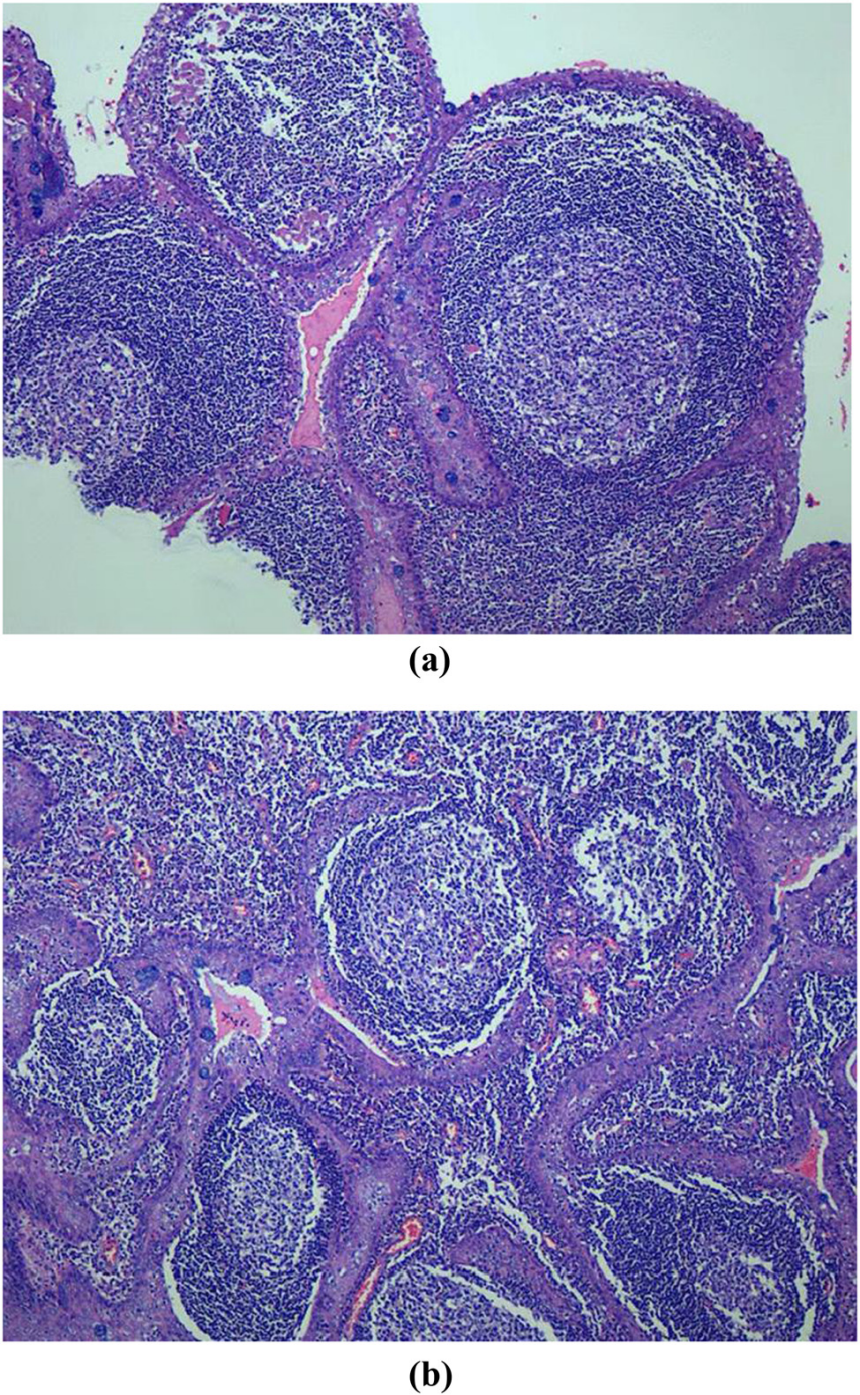
Pathology results of the EUS-FNA operation of abdominal space occupying lesion. The pathology of the needle biopsy shows LEC.

All patients were finally confirmed by pathology results, as shown in Figure 4.

Different treatment plans were selected according to personal situation and patients’ own choice, including surgical treatment (156, 77.6%) and non-surgical treatments such as sclerotherapy, puncture treatment, radiotherapy (24, 11.9%), and dynamic observation (21, 10.5%). Associated hospitalized costs mainly consisted of medication cost (8,457 ± 1,521) and operation cost (5,112 ± 890). The image test cost (2,773 ± 691) was similar as laboratory test cost (2,516 ± 338). No recurrence was found in surgical treatment patients for up to 1-year follow-up (different time periods: 12 months < *T* ≤ 24 months, 52.2%; 6 months < *T* ≤ 12 months, 41.8%; month < *T* ≤ 6 months, 6.0%) and is shown in Table 2.

**Table 2:** Clinical characteristics of LEC

Clinical characteristics (*n*, %)			
Clinical manifestation	Asymptomatic	Painless mass	Abdominal distension
	174 (86.6%)	24 (11.9%)	3 (1.5%)
Medical/Operation history	Hypertension/diabetes/cerebral infarction	Cholecystectomy/appendectomy/gynecological surgical	Healthy before
	111 (55.2%)	48 (23.9%)	42 (20.9%)
Biochemical results	Normal values	CEA increased	CA125/CA199 increased
	171 (85.1%)	21 (10.4%)	9 (4.5%)
Lesion location	Parotid	Oral	Pancreatic
	141 (70.1%)	48 (23.9%)	12 (6.0%)
Size	<2 cm	2–5 cm	>5 cm
	117 (58.2%)	75 (37.3%)	9 (4.5%)
Imaging characteristics	CT/MRI	EUS/EUS-FNA	PET-CT
	198 (98.5%)	12 (6.0%)	3 (1.5%)
Therapeutic schedule	Surgical treatment	Non-surgical treatment	Dynamic observation
	(156, 77.6%)	(24, 11.9%)	(21, 10.5%)
Associated costs (yuan)	Treatment (Medication/operation)	Images costs	Laboratory costs
	8,457 ± 1,521/5,112 ± 890	2,773 ± 691	2,516 ± 338
Follow-up (*T*)	12 months < *T* ≤ 24 months	6 months < *T* ≤ 12 months	1 month < *T* ≤ 6 months
	105 (52.2%)	84 (41.8%)	12 (6.0%)

## 4 Discussion

LEC is a clinical benign and rare lesion. Its morbidity has increased than previous literature due to clinical widespread usage and development of imageology, including CT, MRI, and EUS [5]. The patients were of all ages, most of them were middle-aged. Consistent with previous research [6], our study indicated that male patients were more common. In this research, the largest number of the patients lived in urban area. Higher education and proactive health-screening may lead to more positive findings.

Almost no typical symptoms could be found of LEC [7]. Our study shows that nearly 90% of the patients had no specific clinical manifestation. Only around 10% of the patients noticed a painless mass on the surface of the body. This means that it is difficult to make an accurate diagnosis of LEC only relying on clinical manifestations. Due to the age distribution, nearly half of the patients had cardiovascular and cerebrovascular disease history. About 20% of the patients were well previously. Laboratory tests lack specificity [8]. Most of the patients had normal biochemical results, while minority had increased value of CEA, CA125, or CA199, which needed to be differentiated from malignant neoplastic disease. The predilection sites were located in parotid, mouth, and pancreas. LEC located at the head or neck may occur alone, as well as occur at the base of the mouth with other types of cysts. PLEC was relatively quite rarer [9]. Different LECs had different focal sizes [10]. Our study indicates that more than half of the focal sizes were smaller than 2 cm. Just less than 5% of the focal sizes were bigger than 5 cm. All patients took image tests after hospitalization, including CT, MRI, and PET-CT. CT scan of LEC showed a slightly lower density, slightly higher than the fluid density. On MRI, LEC emerged as a low signal at T1WI, high signal at T2WI, with a clear boundary of cystic and solid mixed image [11]. Furthermore, all patients with abdominal space occupying lesion were performed EUS/EUS-FNA operation. Because of its safe and efficient feature, EUS-FNA could be a reliable way to obtain pathological diagnosis before surgical operation [12]. EUS-FNA has an extremely high pathological positive rate, especially for pancreatic lesions, such as PLEC [13]. The first choice of therapeutic schedule for LEC should be surgical operation, sclerotherapy and radiotherapy could be considered as well [14]. Due to the own characteristic of LEC [15], combined with individual situation, dynamic observation is another feasible selection for LEC patients. Considering the patient’s age, basic diseases, and their willingness to receive treatment, doctors informed the patients of possible treatment options and related prognosis. Therefore, the patients could make personal choices based on their own circumstances (including financial status, family conditions, etc.). At present, for LEC patients, our study found that the largest number of hospitalized associated costs were medication and operation costs. Meanwhile, image costs were similar to laboratory costs. On the one hand, no recurrence was found in surgical treatment and non-surgical treatment patients for up to 24 months follow-up. Unfortunately, nearly 6% of the patients were only followed up for no more than half a year for various reasons (such as contact information or address changed) [16]. On the other hand, no lesion became significantly larger (lesion diameter increased more than 10%) in dynamic observation patients [17].

In general, this study has retrospectively analyzed all the clinical aspects including demographic data, clinical characteristics, and prognosis. It comprehensively showed all the clinical features of this disease. However, the pathogenesis of the disease needs further research to be revealed. Use prospective multi-center studies to further clarify the pathogenesis of LEC. For example, which specific populations are susceptible? What are the direct causes of the disease? How infection or immune dysfunction affects the disease? In the meantime, our research findings from a single-center retrospective study has potential limitations of generalizing findings (such as biased sampling profile, etc.). Multi-center prospective study should be conducted in the future.

To sum up, LEC is a rare and benign lesion, which is mostly located at parotid and oral, rarely located at pancreas. No typical symptoms could be found. EUS-FNA could be a reliable way to obtain pathological diagnosis. LEC could be cured by surgical resection with no recurrence.
